# Dual-modality optical coherence tomography and frequency-domain fluorescence lifetime imaging microscope system for intravascular imaging

**DOI:** 10.1117/1.JBO.25.9.096010

**Published:** 2020-09-30

**Authors:** Xi Chen, Wihan Kim, Michael J. Serafino, Zheng Tan, Javier A. Jo, Brian E. Applegate

**Affiliations:** a Texas A&M University, Department of Biomedical Engineering, College Station, Texas, United States; b University of Southern California, Caruso Department of Otolaryngology–Head & Neck Surgery, Los Angeles, California, United States; c University of Oklahoma, Department of Electrical Engineering, Norman, Oklahoma, United States; d University of Southern California, Department of Biomedical Engineering, Los Angeles, California, United States

**Keywords:** endoscopy, fluorescence spectroscopy, optical coherence tomography, fiber optic applications, atherosclerosis

## Abstract

**Significance:** Detailed biochemical and morphological imaging of the plaque burdened coronary arteries holds the promise of improved understanding of atherosclerosis plaque development, ultimately leading to better diagnostics and therapies.

**Aim:** Development of a dual-modality intravascular catheter supporting swept-source optical coherence tomography (OCT) and frequency-domain fluorescence lifetime imaging (FD-FLIM) of endogenous fluorophores with UV excitation.

**Approach:** We instituted a refined approach to endoscope development that combines simulation in a commercial ray tracing program, fabrication, and a measurement method for optimizing ball-lens performance. With this approach, we designed and developed a dual-modality catheter endoscope based on a double-clad fiber supporting OCT through the core and fluorescence collection through the first cladding. We varied the relative percent of UV excitation launched into the core and first cladding to explore the potential resolution improvement for FD-FLIM. The developed catheter endoscope was optically characterized, including measurement of spatial resolution and fluorescent lifetimes of standard fluorophores. Finally, the system was demonstrated on fresh *ex vivo* human coronary arteries.

**Results:** The developed endoscope was shown to have optical performance similar to predictions derived from the simulation approach. The FLIM resolution can be improved by over a factor of 4 by primarily illuminating through the core rather than the first cladding. However, time-dependent solarization losses need to be considered when choosing the relative percentage. We ultimately chose to illuminate with 7% of the power transmitting through the core. The resulting catheter endoscope had 40-μm lateral resolution for OCT and <100  μm lateral resolution for FD-FLIM. Images of *ex vivo* coronary arteries are consistent with expectations based on histopathology.

**Conclusions:** The results demonstrate that our approach for endoscope simulation produces reliable predictions of endoscope performance. Simulation results guided our development of a multimodal OCT/FD-FLIM catheter imaging system for investigating atherosclerosis in coronary arteries.

## 1 Introduction

Atherosclerosis is the leading cause of morbidity and mortality in the United States.^1^^,^^2^ It is characterized as a systemic, progressive disease process in which the arterial wall thickens through a process of inflammation, oxidative stress, and dyslipidemia.^3^^,^^4^ These arterial plaques may rupture leading to thrombosis and occlusion of the vessel and ultimately, in myocardial infarction, stroke, or limb injury.^5^ Future development of treatment for atherosclerosis will depend on a more detailed understanding of plaque development, including morphological and biochemical information.

The predominant optical intravascular imaging approach, optical coherence tomography (OCT) has been shown to be capable of providing high-resolution structural images revealing the morphological components of plaque in humans.^3^ However, it cannot provide biochemical composition including the relative content of lipoproteins.^6^^–^^8^ Raman spectroscopy can distinguish lipid from necrotic cores, but it requires long measurement times due to weak Raman signal intensity and cannot be utilized for intravascular imaging as a standalone modality.^9^^,^^10^ On the other hand, fluorescence imaging of endogenous fluorophores can provide biochemical information at rates compatible with intravascular imaging.^11^ In particular, fluorescence lifetime imaging microscopy (FLIM) is robust to fluorescence intensity fluctuations and able to measure the relative concentration of lipids and collagen.^12^^–^^14^ The measure and identification of lipoproteins is important for finding so-called “vulnerable” plaques that are prone to rupture causing a sudden coronary event.

There has been growing interest in the development of dual-modality imaging systems for characterizing atherosclerotic plaques. Dual-modality intravascular OCT/FLIM^15^^,^^16^ systems have been developed based on swept-source OCT and time-domain FLIM. Here, we describe a dual-modality intravascular OCT/FLIM system based on swept-source OCT and frequency-domain FD-FLIM. Frequency-domain FLIM offers some advantages, for instance improvement of spatial resolution by propagating excitation light inside the smaller inner core of a double-clad fiber (DCF). This approach is very difficult with time-domain FLIM because the high pulse energies within the inner core can cause fiber solarization damage^17^^–^^19^ and the production of strong stimulated Raman bands.^20^ Furthermore, FD-FLIM is typically less expensive to implement than the comparable time-domain systems we have built in the past,^21^ because we can use relatively inexpensive diode lasers for excitation and avalanche photodiodes for fluorescence detection.^22^

## 2 Materials and Methods

### 2.1 Simulation and Validation of Ball Lens Endoscope FLIM Illumination Performance

**Fig. 1:**
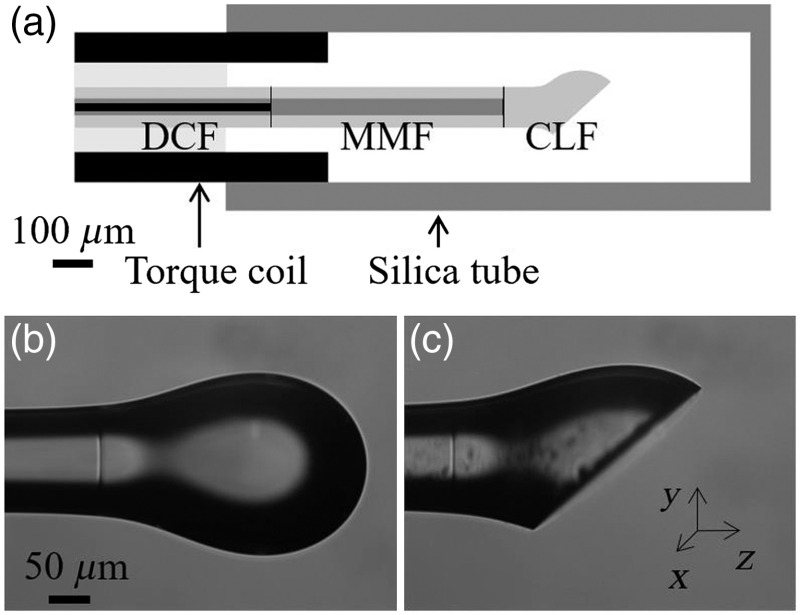
(a) Schematic drawing of the catheter. Heated and shaped CLF fusion spliced with an MMF and then with a DCF, and the fiber was glued with torque coil and silica tube. Torque coil’s i.d. is 270  μm and o.d. is 450  μm. Silica tube’s i.d. is 530  μm and o.d. is 620  μm. Scale bar: 100  μm. DCF, double-clad fiber; MMF, multimode fiber; CLF, coreless fiber. (b) Side view of the fabricated ball lens before polishing. Heated and shaped CLF fusion spliced to an MMF [DCF was not shown due to small field of view (FOV) of the machine]. Scale bar: 50  μm. (c) Side view of the ball lens after polishing by customized fiber polishers. The polishing angle is 41 deg.

Prior to fabrication, we simulated the performance of the endoscope via the optical ray-tracing program OpticStudio (Zemax) to optimize the ball lens design. A polished ball lens at the end of fiber endoscope is used to focus and reflect light, as shown in Fig. 1. Simulation is important to minimize manufacturing time by establishing a preferred shape for achieving target optical properties with the partial ellipsoid lens. In the simulation, the 1.3-μm OCT light was considered to be completely contained within the single mode core of the DCF. The light exiting the core was simulated with Gaussian beam propagation after the ball lens to predict beam details for both illumination and collection, which is more accurate for side-viewing, noncircularly symmetric, and nonparaxial ball lenses than the ABCD matrix method.^23^^,^^24^ The ball lens shape was optimized according to a merit function considering optical performance and tolerance analysis. Once manufactured, it was straightforward to directly measure the output of the ball lens using a beam profiler and 1.3-μm laser diode illumination for comparison.

FLIM excitation was considered to transmit through both the core and the inner cladding of the DCF. We simulated the beam profile including spatial mixing within the multimode fiber (MMF). Mixing of spatial modes within the MMF leads to rapid variations in the intensity profile exiting the fiber, which poses a problem both for adequate simulation and finally comparison with measured ball lens performance. To overcome this issue, we sought to identify an intensity profile that could be modeled in OpticStudio as well as reliably generated and measured using standard optical techniques. We chose the super-Gaussian profile that approximates a top-hat profile with Gaussian tails on either side, which could fit the beam profile in both near field and far field. The intensity profile is $\;I(r)=I_{0}\;\exp[-2{\left(r/w_{m}\right)}^{n}]$, where r is the radial dimension, $w_{m}$ is the beam waist, and n is the order of the super-Gaussian. A model intensity profile was generated by measuring the intensity profile from the core and inner claddings of a segment of DCF using a beam profiler. The angular distribution was fit to a super-Gaussian using a series of five far-field measurements at intervals of 508  μm. These measurements were done nine times to test for reproducibility, each time removing and replacing the fiber. The fit of the super-Gaussian proved to be robust to fiber bending and readily reproducible with a standard deviation of 1% or less for the nine measurements at the five intervals (n=45). We used this to approximate the intensity profile impinging on the coreless fiber (CLF), which was modeled based on the source-radial using nonsequential mode with OpticStudio.

The final piece needed for simulation is the ball-lens design itself. The ball-lens was drawn in Solidworks with the desired ellipsoidal shape and imported into OpticStudio. Using the approaches described above, the performance for both OCT and FLIM could be modeled. Likewise, since we directly used the super-Gaussian intensity, we measured from the DCF to simulate the ball-lens, thus we could reliably test performance of the ball-lens after fabrication.

### 2.2 Design of Ball Lens Endoscope

A fiber preparation and splice workstation (FFS2000, Vytran) was used for endoscope fabrication. The 1.55-m OCT/FLIM endoscope was constructed starting with a DCF, SM-9/105/125-20A by Nufern. The DCF was fusion spliced to an MMF (FG105UCA, Thorlabs). The MMF was cleaved to a precise length and then fusion spliced to a CLF (HCF-125-47, Coractive). Following splicing, the CLF was cleaved to 1250-μm length, heated and shaped into an ellipsoid ball with a total CLF length of 632  μm and predetermined semiprinciple axes (140.6 and 123.1  μm) in the tungsten filament furnace of the workstation, shown in Fig. 1(b). A schematic drawing is shown in Fig. 1(a). The ellipsoidal shape was designed to compensate for the astigmatism caused by the toroidal shape of the catheter sheath and the silica tube used to cover and protect the fiber endoscope. The distal ball lens was polished with an angle of 41 deg using a commercial fiber polisher (69-3000-160, Buehler) with custom modifications to enable ball lens polishing at precise angles for side viewing endoscopes. The 41-deg angle was chosen so that total internal reflection would occur at the polished glass–air interface, hence obviating the need for reflective coatings on the polished surface. In addition to the angle, precise control of the polishing depth from an unpolished ball is necessary to achieve good performance. Images taken before and after polishing are shown in Figs. 1(b) and 1(c).

**Fig. 2:**
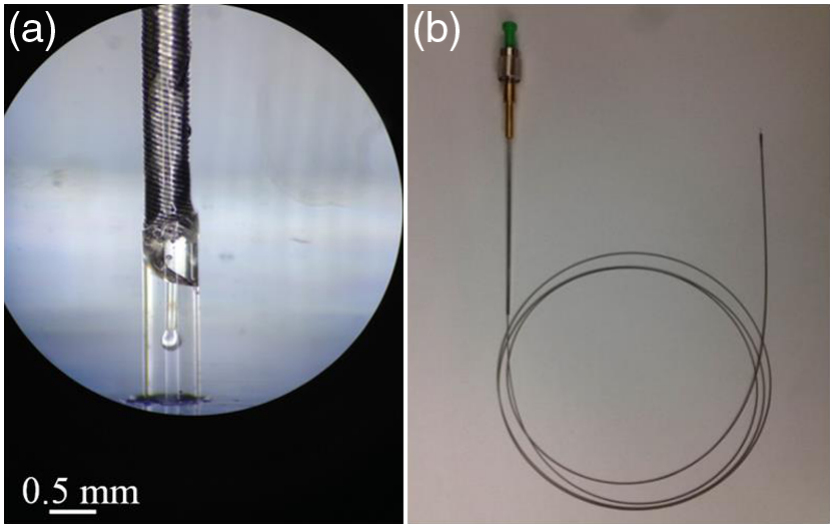
(a) Ball lens enclosed within silica tube. Both ends of the silica tube were sealed by epoxy. (b) Image of a catheter endoscope, the proximal end was connectorized by FC/APC connector.

The fiber endoscope was then enclosed in a customized torque coil (ASAHI INTECC) with high-speed torque transmission and high flexibility. The outer diameter (o.d.) of the torque coil is 450  μm and the ID is 270  μm. The proximal end was connectorized with a custom FC/APC connector and strain relief boot, mating to a custom broadband fiber optic rotary joint.^16^ The distal end was covered by a laser-cut silica tube (TSP530620, Polymicro) with an outer diameter of 620  μm and an inner diameter of 530  μm, shown in Fig. 2. The silica tube serves both to protect the ball lens and preserve the air–glass interface at both the flat polished and curved surfaces of the endoscope even when submerged. The i.d. of the silica tube was chosen so that the torque coil could fit inside, and therefore, make a strong bond between the two. Using a smaller diameter for the silica tube would unnecessarily increase the astigmatism introduced by the tube. The fiber endoscope was inserted into a custom designed flexible housing with an i.d. of 0.812 mm and o.d. of 1.066 mm.

### 2.3 OCT/FLIM Instrumentation

**Fig. 3:**
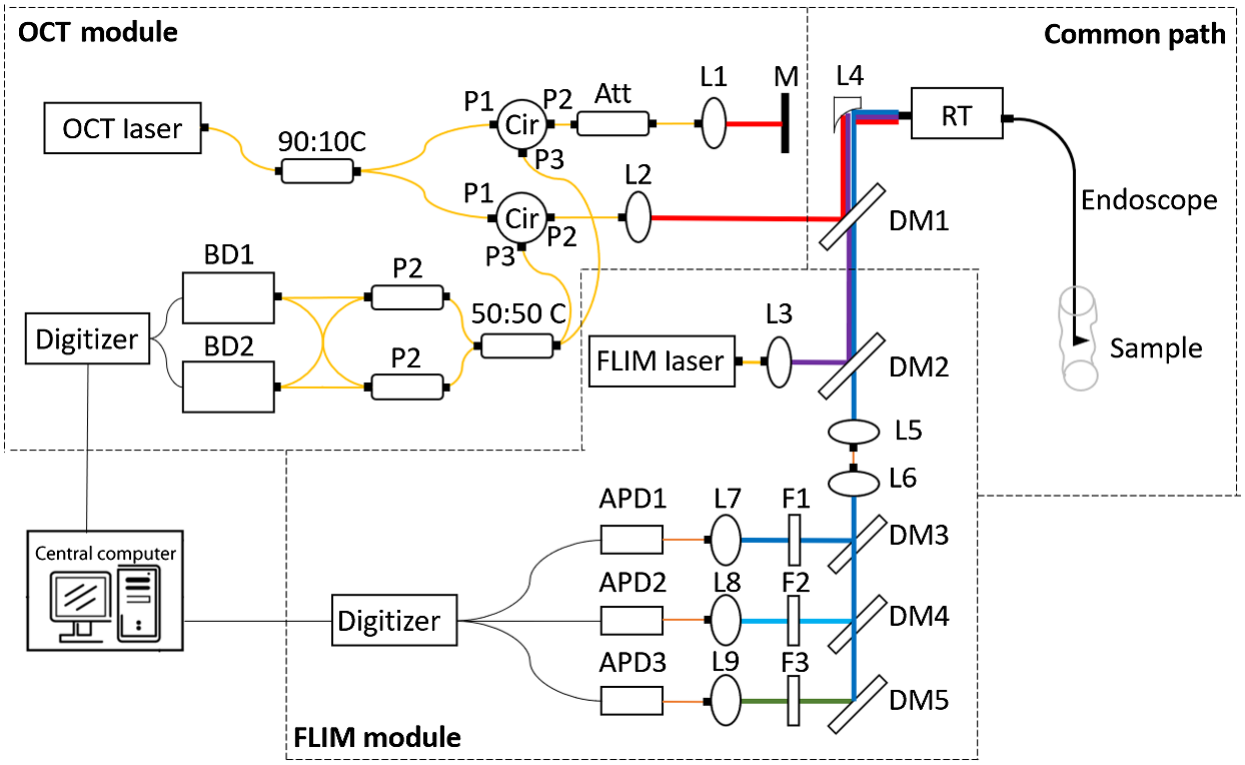
Schematic diagram of the dual-modality imaging system, including OCT module, FLIM module, and common path. L1 to L3, L7 to L9, free-space collimation and coupling lenses; L4, reflective collimator; L5, L6, fiber-connected collimation and coupling lenses; M, mirror; DM1 to DM5, dichroic mirrors; F1 to F3, bandpass filters; PL, polarizer; BD, balanced detectors; Cir, circulators; P1 to P3, circulator ports 1 to 3; APD1 to APD3, avalanche photodiodes; RT, rotary joint. Orange line indicates MMF fiber transmission. Yellow line indicates SMF transmission. Other thick colored lines indicate free-space transmission. Black thin line indicates electrical line.

A schematic diagram of the dual-modality imaging system is shown in Fig. 3. The diagram can be divided into the OCT module, FLIM module, and common path. The common path refers to the portion of the optical system that is common to both OCT and FLIM, i.e., the respective beams are copropagating.

#### 2.3.1 OCT module

A fiber-based swept-source OCT system was implemented as the OCT module. The light source was a fiber-coupled wavelength-tunable laser (ESS-1310 nm-100 kHz, Exalos) with a center wavelength of 1310 nm and a spectral bandwidth of 100 nm. The light exited the laser via a single-mode fiber (SMF-28e, Corning) and was coupled into a 90:10 fiber coupler, directing 90% to the sample arm and 10% to the reference arm. The sample arm light was directed to a circulator and then on to the rotary joint and endoscope, as described below. The backscattered light exiting port 3 (P3) of the circulator was recombined with the reference arm (P3) in a standard 2×2 (50:50 C) fiber coupler.

It is well known that rotating catheter endoscopes, as described here, generate fluctuations in the light polarization in the sample arm. In order to overcome this, polarization diverse detection was implemented.^25^ The two outputs of the 2×2 coupler (50:50 C) that combined the reference and sample arms were each coupled into a polarizing 1×2 coupler (P2, PBS-31-P-2-L-3-1, General Photonics). The s-polarization from each coupler was input into one balanced photodiode detector and the p-polarization to the other. In order to have equal reference s- and p-polarization intensities, the reference arm light was adjusted with a set of polarization paddles. The signal from the two balanced photodiode detectors (WL-BPD600MA, Wieserlabs UG) were digitized at 200 MS/s simultaneously using an acquisition card NI-5772 (800 MS/s, 12-Bit, 2-Channel Digitizer Adapter) with an inline field-programmable gate array (NI, PXIe-7966). The signal from a reference interferometer was used to calibrate the laser sweep once before each imaging session before the experiment. The A-line was taken as the vector sum of the two channels, i.e., $s=\sqrt{s_{1}^{2}+s_{2}^{2}}$, where $s_{n}$ is the amplitude of the corresponding FFT. This served to minimize the modulation of the A-line amplitude due to polarization variations. The sample arm of the interferometer will be described in Sec. 2.3.3.

#### 2.3.2 FLIM module

The FLIM module was similar to that described in the previous paper.^22^ A fiber-based diode laser (iBeam-SMART-375-S, Toptica) with a modulation of up to 250 MHz was used as an excitation light source for FLIM. The excitation/emission light copropagates with the OCT light as discussed below, in Sec. 2.3.3. The fluorescence emission exiting the common path was directed into the multispectral detection module by a dichroic mirror (DM2) (FF376-Di01-25x36, Semrock). A short, semiarbitrary length of 200-μm MMF (FG200UEA, Thorlabs) was used to transmit light to the fluorescence detection system. After transmitting through the ∼0.4  m of MMF, the emission was directed into a multispectral detection system with a set of dichroic mirrors (D3, ZT405rdc; D4, ZT488rdc; D5, T560LPXR, Chroma) and bandpass filters (F1: ZET405/20x, Semrock; F2: FF-1-440/40; F3, FF03-525/50, Chroma) to divide the emission into three bands (Ch1 405/20, Ch2 440/40, and Ch3 525/50). Each channel had its own preamplifier (ZFL-500LN+, Mini Circuits) and avalanche photodiode (APD) (C12702-11, Hamamatsu) detector. Outputs from the three APDs were digitized at 250 MS/s with 14-bit resolution and processed on an FPGA (NI5761 and PXIe7962, National Instruments).

The digitized time-domain fluorescence signal was processed in the following way. A discrete Fourier transform was calculated at 1.25, 20.83, 41.67, 62.50, and 83.33 MHz. Then, a background fluorescence signal was subtracted before computing the phase and modulation lifetimes. The background was measured by recording the FLIM image without a sample (i.e., an air sample) and taking the mean of the entire image. To compensate for the system response, fluorescence from POPOP was recorded as a standard (τ=1.35  ns) and used to calculate correction factors, which were applied directly to the phase and modulation lifetimes. The discrete Fourier transform (DFT) was performed on the FPGA while all other processing was performed on the host computer.

#### 2.3.3 Common path

The OCT sample arm light and FLIM excitation light were combined by a dichroic mirror DM1 (FF700-SDi01-25x36, Semrock) and then coupled into a DCF with an off-axis parabolic reflective collimator, L4 in Fig. 3 (RC04APC-F01, Thorlabs). The parabolic reflective collimator was chosen to mitigate chromatic aberration over this extremely wide bandwidth of light source (375 to 1360 nm). The coupling efficiency was 79% for 1310-nm light into the single mode core and 87% for the 375-nm FLIM excitation light into the inner cladding. The fiber endoscope, described above, was connected to an ultrabroadband lensless fiber optic rotary joint.^26^ The lensless rotary joint achieved very low insertion loss and noise variance (<0.2  dB) even at high rotational velocity (8800 revolution per minute, rpm).

### 2.4 Tissue Preparation and Imaging

Human coronary artery segments were obtained from autopsy cases within 5 days of the time of death according to a protocol approved by the Texas A&M University Institutional Review Board. The arterial segment was imaged with helical beam scanning by rotating the endoscope inside the lumen of the artery while pulling the endoscope back. Immediately after imaging, each segment was ink marked for correlation with histopathology, fixed in 10% formalin, and sent for histopathology analysis. Each imaged artery segment was consecutively sectioned every 1 mm. The sections were stained with Movat pentachrome and CD68. Movat stains collagen, elastin, muscle, mucin, and fibrin in tissue sections and is commonly used for coronary arteries. CD68 stains macrophages.

## 3 Results and Discussion

### 3.1 Solarization and Absorption Losses in the Fiber Endoscope

Transmission loss due to absorption and solarization are important when coupling high power UV light into optical fibers as we do for FLIM excitation. As has been reported previously, high intensity pulses suffer greater losses due to two-photon absorption.^17^^,^^27^ Two-photon absorption has been shown to be the dominant process for the formation of radiation-induced defects in silica material.^18^ Similarly, solarization and thermal cumulative effects are known to become significant for high power UV transmission through the core.^17^^,^^19^^,^^28^ For our system, the intensity of light can be up to $0.1\;\mathrm{MW}/{\mathrm{cm}}^{2}$ if transmitting through the core. Therefore, both processes should be considered carefully since they can degrade the fiber transmission and affect the optical performance.

**Fig. 4:**
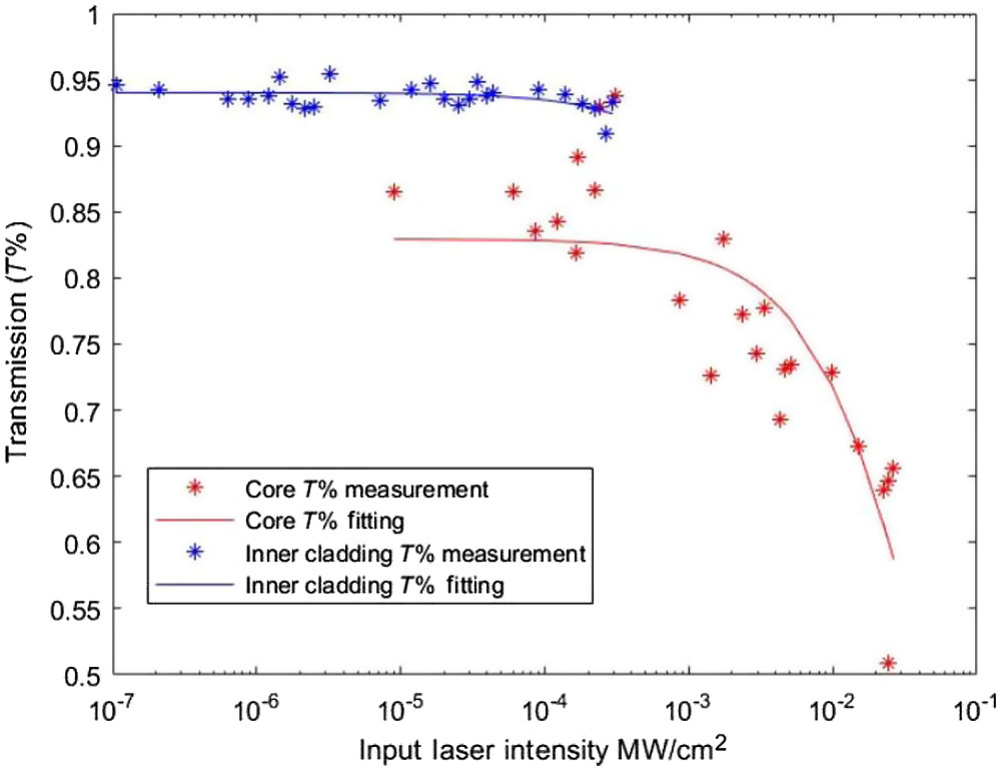
One-photon absorption and two-photon absorption at 375 nm. Curves are fitting of Eq. (1). Transmission of the inner cladding is measured based on 40-cm DCF, and transmission of the core is measured based on 59.5-cm DCF.

The fiber transmission, considering one- and two-photon absorption, can be described as

$$T^{-1}=(1+\beta )\exp(\alpha_{0}l)-\beta ,$$ (1)

where T is the fiber transmission, $\alpha_{0}$ is the one-photon absorption coefficient, $\alpha_{1}$ is the two-photon absorption coefficient, $\beta =\alpha_{1}I_{\text{input}}/\alpha_{0}$ is the propagation constant, and l is the fiber length.^17^ Using this equation, we measured the one- and two-absorption coefficients of the core and inner cladding by measuring the fiber transmission as a function of the length of the fiber using the cut-off method. The output power of a known length of DCF patch cable was measured. A segment was then removed and the outpower measured again. This process was repeated to build up the set of data shown as the asterisk in Fig. 4. Pulses at 375 nm and 500 ms duration were launched into the core and inner cladding to generate the two sets of data. A 500-ms pulse was chosen to make sure solarization was minimal with negligible impact on absorption measurement accuracy. A fit of the data using Eq. (1) is shown as solid lines in Fig. 4. The resulting one-photon absorption and two-photon absorption coefficients were $3.14\times 10^{-3}\;{\mathrm{cm}}^{-1}$ and $2.88\times 10^{-1}\;\mathrm{cm}/\mathrm{MW}$ in the core and $1.54\times 10^{-3}\;{\mathrm{cm}}^{-1}$ and 1.50 cm/MW in the inner cladding.

**Fig. 5:**
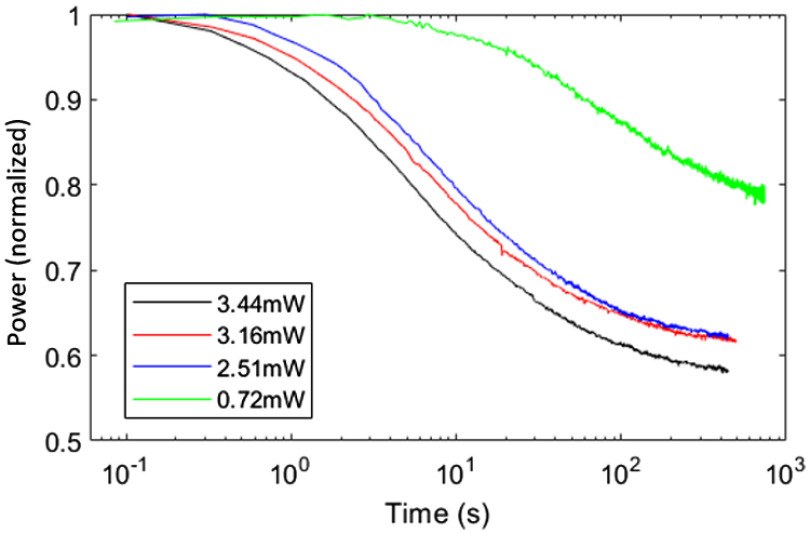
Solarization rate of DCF core. The DCF core was solarized when the power was higher than 0.7 mW. Solarization rate increases with power.

Ultraviolet radiation degrades the fiber transmission (solarization), especially in high-OH fiber. The solarization rate of the DCF is important for our application since we want to be confident that the optical properties of the system are not changing over the course of an imaging session. In order to measure the solarization rate of the core, we used the following technique. An SMF (SMF28e, Corning) patch cable was presolarized by illumination with 50 mW of 375 nm light so that the transmission was stable and solarization recovery did not happen after 1 h. This patch cable was connected to a 1.55-m DCF patch cable so that the light from the core of the SMF was reliably transmitted to the core of the DCF. The output power from the DCF was then measured as a function of time to produce the curves in Fig. 5. As expected, the solarization rate increases when the input power to the DCF is increased. We tried a similar experiment on the inner cladding; however, we were not able to measure any change in transmission over the time course of the experiment, hence no significant solarization. We also measured the absorption coefficients using the same cut-off method noted above after the solarization experiment. The one-photon coefficient for the core more than doubled to $7.26\times 10^{-3}\;{\mathrm{cm}}^{-1}$, while the one-photon coefficient remained approximately the same for the inner cladding, $1.61\times 10^{-3}\;{\mathrm{cm}}^{-1}$. The small increase in the one-photon absorption can be explained by the fact that the measurements on the inner cladding include light that propagates through the core. The two-photon coefficients cannot be measured because the 50-mW incident power cannot induce a strong enough intensity for two-photon coefficients measurements when the one-photon absorption is so high.

It would be advantageous to illuminate the sample through the core in order to achieve ∼4× higher spatial resolution than illumination through the inner cladding. However, given that an imaging session will likely require the illumination of the catheter for 10s of seconds, if we excite through the core, we will lose about 30% to 40% of the excitation light due to solarization. Since collection of the fluorescence is largely dependent on the inner cladding transmission, it should not be significantly impacted. Hence given a bright enough light source, it should be possible to illuminate through the core to gain higher spatial resolution. More generally, if longer wavelength illumination can be used, it would also mitigate the solarization issue.

### 3.2 FLIM Resolution and Mechanical Testing

Given that we were testing a new system with imaging times that would lead to strong solarization and that DCF have been fabricated to guide UV either entirely through the core or the inner cladding,^29^ we chose to primarily illuminate through the inner cladding. We aligned the optical system to adjust the output power ratio between core (7%) and the inner cladding (93%) for a trade-off between spatial resolution and output power.

**Fig. 6:**
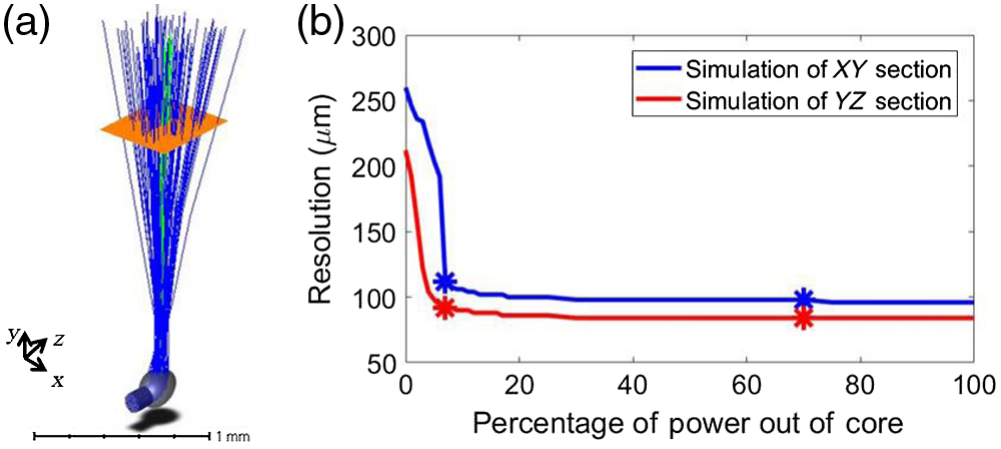
OpticStudio simulation FLIM endoscope performance. (a) 3-D layout of OpticStudio simulation. The gray object is the fabricated ball lens imported from SolidWorks. Blue rays are random layout rays launching from the inner cladding, and green rays are random layout rays launching from the core. The orange plane represents the receiver that was translated along the beam bath to extract the resolution plotted in (b). (b) Simulation of resolution of XY section and YZ section at 1 mm from the center of the fiber with different power transmitting out of the core. Asterisks indicate simulated resolution with two scenarios mentioned in the text.

We optimized the mode mixing in the DCF to generate a super-Gaussian profile. We found this to be stable even when the endoscope fiber is rotating rapidly. The stability of the super-Gaussian output gave us confidence that we could expect a similar intensity profile at the interface of the CLF and the DCF in the endoscope. The intensity profiles from the core and the inner cladding were modeled based on two source-radial sources. The collection efficiency was also modeled for resolution estimation. The three-dimensional (3-D) layout from OpticStudio including rays from the core (green) and inner cladding (blue) is shown in Fig. 6(a). The plane (orange) represents the receiver where the beam profile was simulated. The resolution at 1 mm from the center of the fiber is shown in Fig. 6(b). The XY section FWHM resolution and YZ section FWHM resolution were 99 and 88  μm, respectively, while 70% [* in Fig. 6(b)] power was transmitting out of the core and 30% power was transmitting out of the inner cladding. Under our planned experimental conditions, i.e., 7% power transmitting out of the core, the XY section resolution and YZ section resolution were 111 and 99  μm, respectively [* in Fig. 6(b)]. Nominally, the resolution can be tuned by adjusting the relative power transmitting through the core and the inner cladding.

**Fig. 7:**
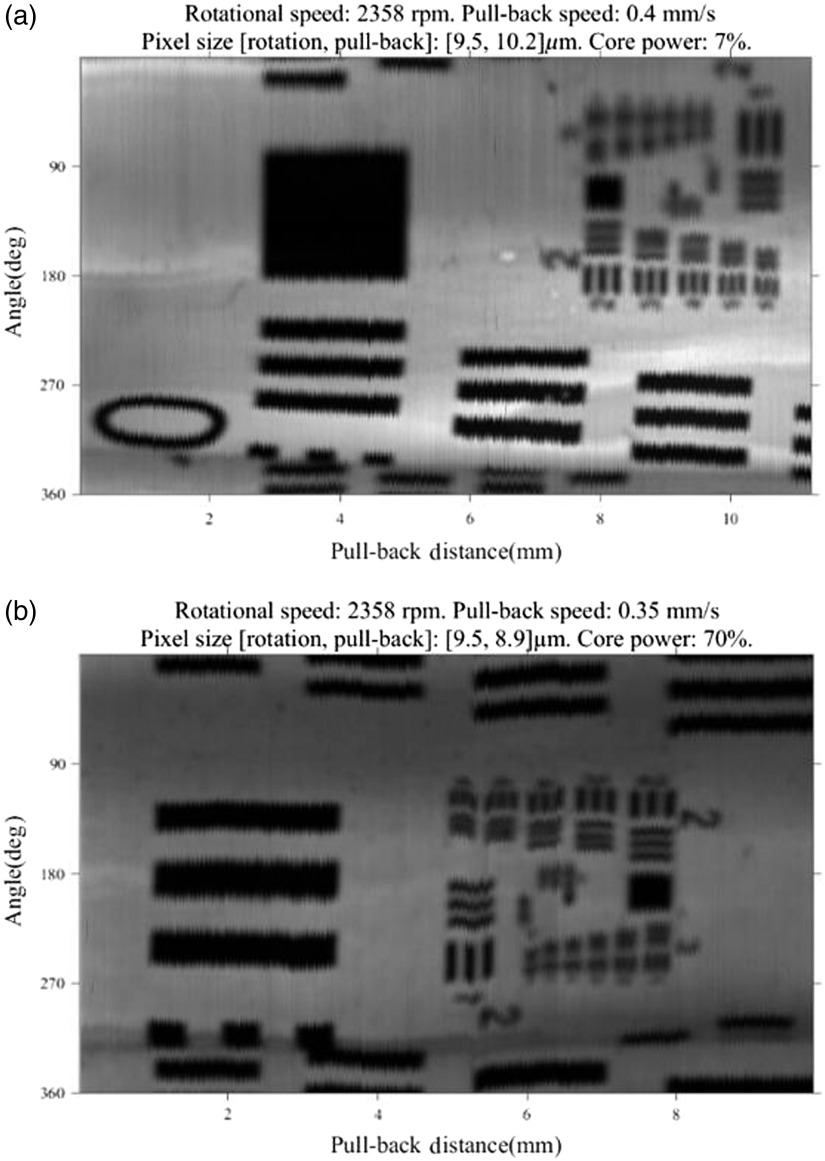
Pullback fluorescence intensity images of resolution targets. (a) The bars show up dark in this gray scale image, and we can clearly differentiate bars with the width of 99  μm radially and 111  μm longitudinally while 7% power transmitting out of the core. (b) We can clearly differentiate bars with the width of 88  μm radially and 99  μm longitudinally while 70% power transmitting out of the core.

In order to confirm the resolution of the endoscope under realistic imaging conditions, a resolution target (53-715, Edmund Optics) was wrapped around a silica tube with 1-mm i.d. and 2-mm o.d. The white paper has high fluorescence while the black ink has low fluorescence. The fluorescence intensity maps were used to define FLIM spatial resolution. The tube was filled with phosphate-buffered saline (PBS) and the endoscope inserted. Several pullback images were acquired with representative intensity maps shown in Fig. 7. In Fig. 7(a), the endoscope was rotated at 2358 rpm and pulled back at a rate of 0.4  mm/s. Under these conditions, the image sampling is 9.5  μm along the radial dimension and 10.2  μm along the longitudinal dimension at the surface of the resolution target. The fluorescence is attenuated in areas with ink hence, the bars show up dark in this gray scale image. We can clearly differentiate bars with a width of 111  μm (group 2 element 2) in the rotation direction and 99  μm (group 2 element 3) in the longitudinal direction. In Fig. 7(b), the endoscope was rotated at 2358 rpm and pulled back at a rate of 0.35 mm/s. Under these conditions, the image sampling is 9.5  μm along the radial dimension and 8.9  μm along the longitudinal dimension at the surface of the resolution target. We can clearly differentiate bars with a width of 99  μm (group 2 element 3) in the rotation direction and 88  μm (group 2 element 4) in the longitudinal direction. Experimentally, we expect deviations from the simulation based on the fact that we are imaging through a silica tube which has a higher refractive index than saline, and therefore, should lead to a smaller beam waist. Likewise, the endoscope is free to deviate from the center of the silica tube during rotation.

### 3.3 Fluorescence Lifetime Imaging

In order to validate the temporal and spectral calibration of the catheter imaging system, we acquired images of solutions of well-characterized fluorophores. The distal end of the catheter (∼1  cm) was immersed in a solution of 0.1-mM POPOP, 1-mM NADH, and 1-mM FAD, respectively. The emission was spectrally resolved into three channels centered at 405, 440, and 525 nm. A spectral intensity calibration to compensate for wavelength-dependent losses was performed on the three spectral bands based on the measured POPOP spectrum, known transmission curves of filters and dichroic mirrors, and the POPOP spectrum^13^ from the literature. The calculated lifetimes were 1.35±0.04  ns for POPOP (note, also used as standard), 0.56±0.23  ns for NADH, and 2.14±0.40  ns for FAD. The literature values for the average lifetime of POPOP, NADH, and FAD are 1.38±0.01,^30^ 0.44,^31^ and 2.07 ns,^32^ respectively. The results agree with literature values within one standard deviation of our measurement except POPOP, which differed by slightly more than one standard deviation. We consider this is to be good agreement.

### 3.4 *Ex Vivo* Human Coronary Artery and Histology Analysis

**Fig. 8:**
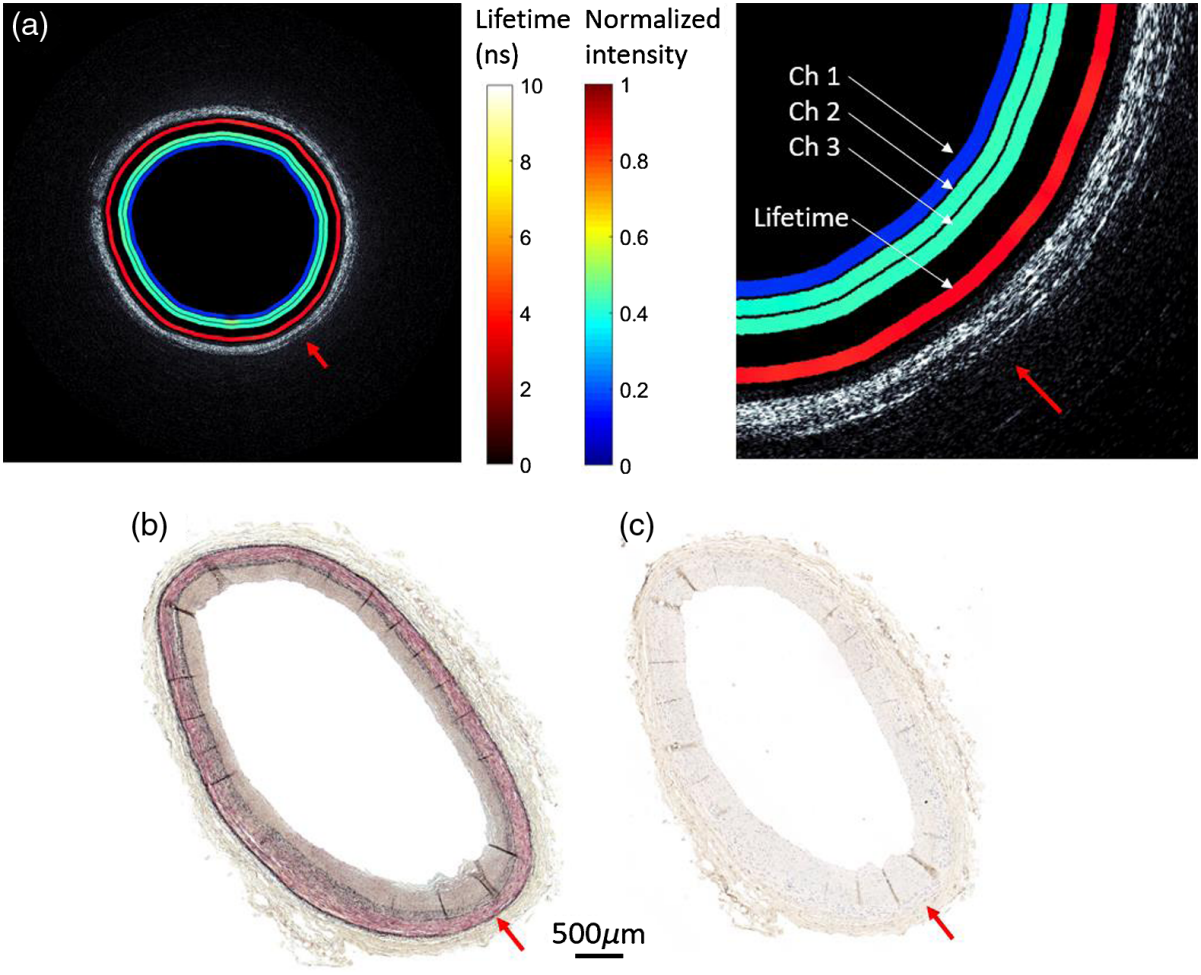
(a) OCT/FLIM cross-sectional image of *ex vivo* of human coronary artery obtained from autopsy. The OCT is rendered in gray scale. The four color rings on the inside of the lumen are from inner to outer normalized intensity maps from channels 1, 2, 3 and lifetime map (channel 3 at 40 MHz). (b) Histopathology result (Movat staining). (c) Histopathology result (CD68 staining). Red arrow provides a point of reference for all four images.

**Fig. 9:**
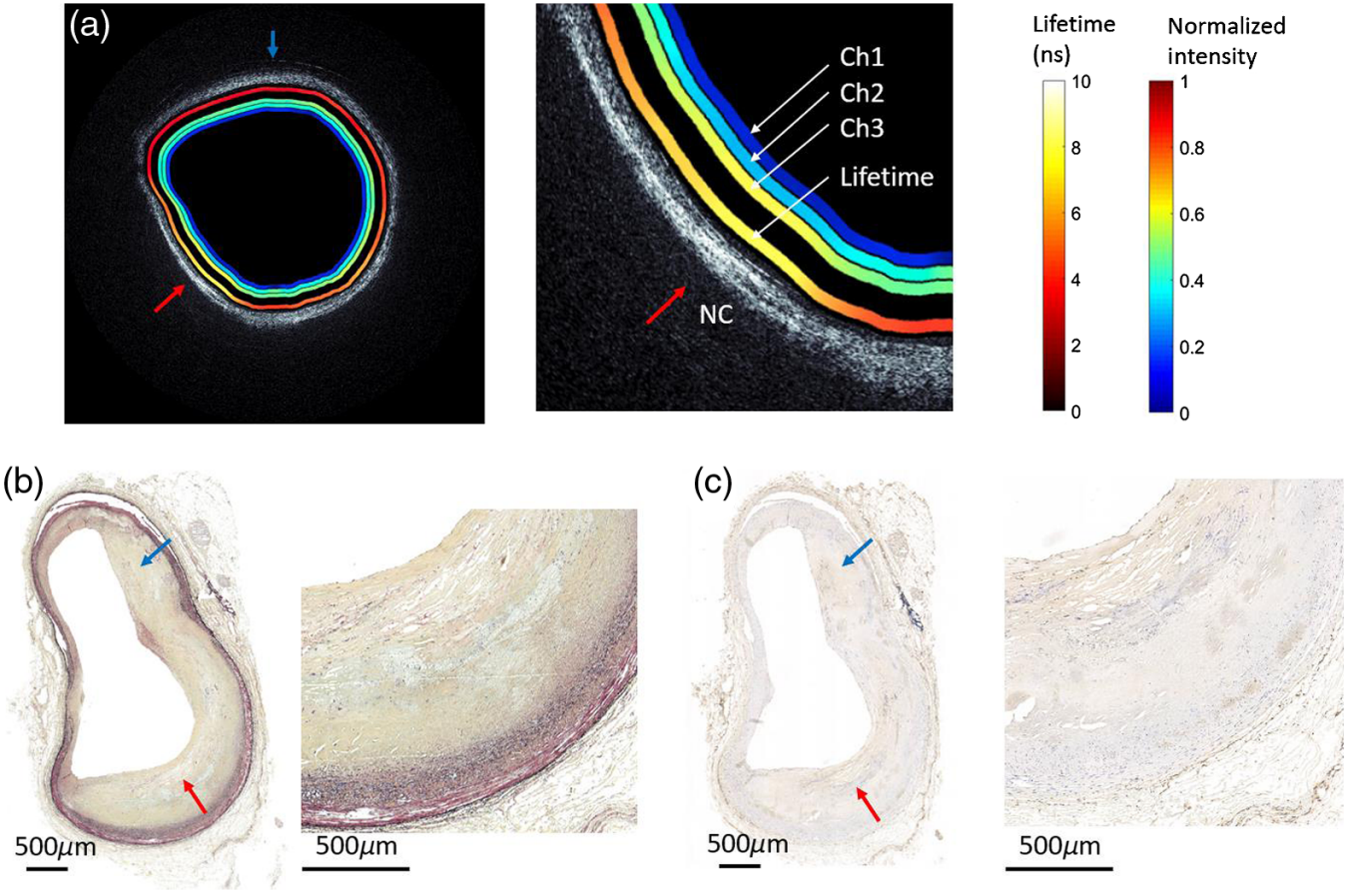
(a) OCT/FLIM cross-sectional image of *ex vivo* of human coronary artery obtained from autopsy. The OCT is rendered in gray scale. The four color rings on the inside of the lumen are from inner to outer normalized intensity maps from channels 1, 2, 3 and lifetime map (channel 3 at 40 MHz). (b) Histopathology result (Movat staining). (c) Histopathology result (CD68 staining). Red arrow points at a fibroatheroma with a thick cap and a small necrotic core (shown in zoomed view). Blue arrow points at the thickened intima.

Finally, we imaged a segment of human coronary artery obtained from autopsy. The catheter endoscope was inserted into the flexible catheter which was then placed in the artery lumen. A pullback at 1.87 mm/s was performed at a rotational velocity of 3000 rpm. A representative OCT image and overlapped fluorescence lifetime map are shown in Figs. 8 and 9. After completing imaging, the artery segment was fixed in formalin and sent for histopathology processing.

The histopathology (Fig. 8) indicates a fibrotic plaque rich in collagen and smooth muscle cells around the entire lumen with no observable macrophage infiltration. The intravascular OCT (IVOCT) figure shows a layered architecture, with highly backscattering intima and adventitia and low backscattering media. The intima area is region of thickened collagen, which has lifetime of ~5 ns.

The histopathology of the second artery sample (Fig. 9) shows a fibroatheroma with a thick cap rich in collagen and smooth muscle cell covering a small necrotic (NC) [red arrow in Fig. 9(b)] showing extensive macrophage infiltration [red arrow in Fig. 9(c)]. The region next to the fibroatheroma shows a thickened intima rich in collagen [blue arrow in Fig. 9(b)] and minimal macrophage infiltration [blue arrow in Fig. 9(c)]. The OCT figure overlapped with FLIM lifetime map shows two distinct regions resembling: (a) a region (blue arrow) of thickened collagen (homogeneous IVOCT signal low signal drop-off and fluorescence lifetime ∼5  ns) and (b) a region (red arrow) of combination of collagen and lipid (a fast IVOCT signal drop-off and fluorescence lifetime >6  ns^14^) reflecting the presence of the lipid-rich necrotic core and macrophage.

## 4 Conclusions

In conclusion, we have developed a dual-modality intravascular swept-source OCT and frequency-domain FLIM catheter system to capture both structural and biochemical information on atherosclerotic plaques. We demonstrated a combined simulation and measurement approach for designing ball lens endoscopes that could be applied to a wide range of ball sizes using any available DCF. This approach enables careful optimization of the design before fabrication and allows us to simulate the performance of the endoscope in environments where it is not practical to make direct measures of optical properties, such as the coronary artery with a saline flush. We demonstrated that the resolution of the FLIM system could be tuned by varying the relative power distributed to the core and inner cladding. With UV excitation, this approach is confounded by solarization of the optical fiber, which introduces significant optical loss. This can be overcome by either using a higher power light source to compensate for solarization losses or utilizing longer wavelengths for excitation to avoid solarization.

We have experience developing and using both time-domain and frequency-domain FLIM systems for imaging atherosclerotic plaques. We believe that the frequency-domain approach offers key advantages over time-domain systems. The ability to achieve higher spatial resolution and tune that resolution could enable more detailed images of the plaques. While we did not demonstrate it here, in principle, it should be possible to use higher average power for excitation because the peak power is orders of magnitude lower. This should translate to a higher signal to noise ratio. Finally, in our experience, the system cost is substantially lower for frequency-domain systems^22^ compared to time-domain systems we have developed before.^21^ This is important if the technology is to be translated to the clinic.

## References

[1] Benjamin E. J. et al., “Heart disease and stroke statistics—2018 update: a report from the American Heart Association,” Circulation 137(12), e67–e492 (2018). CIRCAZ 0009-7322 10.1161/CIR.0000000000000558 29386200

[2] Benjamin E. J. et al., “Heart disease and stroke statistics—2017 update: a report from the American Heart Association,” Circulation 135(10), e146 (2017). CIRCAZ 0009-7322 10.1161/CIR.0000000000000485 28122885 PMC5408160

[3] Tearney G. J. et al., “Consensus standards for acquisition, measurement, and reporting of intravascular optical coherence tomography studies,” J. Am. Coll. Cardiol. 59(12), 1058–1072 (2012). JACCDI 0735-1097 10.1016/j.jacc.2011.09.079 22421299

[4] Finn A. V. et al., “Concept of vulnerable/unstable plaque,” Arterioscler. Thromb. Vasc. Biol. 30(7), 1282–1292 (2010). ATVBFA 1079-5642 10.1161/ATVBAHA.108.179739 20554950

[5] Virmani R. et al., “Pathology of the vulnerable plaque,” J. Am. Coll. Cardiol. 47(8 Suppl.), C13–C18 (2006). JACCDI 0735-1097 10.1016/j.jacc.2005.10.065 16631505

[6] Maarek J. M. I. et al., “Time-resolved fluorescence of human aortic wall: use for improved identification of atherosclerotic lesions,” Lasers Surg. Med. 27(3), 241–254 (2000). LSMEDI 0196-8092 10.1002/1096-9101(2000)27:3<241::AID-LSM6>3.0.CO;2-0 11013386

[r7] Arakawa K. et al., “Fluorescence analysis of biochemical constituents identifies atherosclerotic plaque with a thin fibrous cap,” Arterioscler. Thromb. Vasc. Biol. 22(6), 1002–1007 (2002). ATVBFA 1079-5642 10.1161/01.ATV.0000017461.79231.3D 12067911

[8] Marcu L., “Fluorescence lifetime in cardiovascular diagnostics,” J. Biomed. Opt. 15(1), 011106 (2010). JBOPFO 1083-3668 10.1117/1.3327279 20210432 PMC2847934

[9] Dochow S. et al., “Comparing Raman and fluorescence lifetime spectroscopy from human atherosclerotic lesions using a bimodal probe,” J. Biophotonics 9(9), 958–966 (2016). 10.1002/jbio.201500341 27003796 PMC5012915

[10] Cordero E. et al., “*In-vivo* Raman spectroscopy: from basics to applications,” J. Biomed. Opt. 23(7), 071210 (2018). JBOPFO 1083-3668 10.1117/1.JBO.23.7.071210 29956506

[11] Ma D. et al., “Technique for real-time tissue characterization based on scanning multispectral fluorescence lifetime spectroscopy (ms-TRFS),” Biomed. Opt. Express 6(3), 987–1002 (2015). BOEICL 2156-7085 10.1364/BOE.6.000987 25798320 PMC4361450

[12] Jo J. A. et al., “Simultaneous morphological and biochemical endogenous optical imaging of atherosclerosis,” Eur. Heart J.–Cardiovasc. Imaging 16(8), 910–918 (2015). 10.1093/ehjci/jev018 25722204 PMC4592321

[r13] Park J. et al., “Biochemical characterization of atherosclerotic plaques by endogenous multispectral fluorescence lifetime imaging microscopy,” Atherosclerosis 220(2), 394–401 (2012). ATHSBL 0021-9150 10.1016/j.atherosclerosis.2011.10.034 22138141 PMC3264694

[14] Rico-Jimenez J. J. et al., “Automated detection of superficial macrophages in atherosclerotic plaques using autofluorescence lifetime imaging,” Atherosclerosis 285, 120–127 (2019). ATHSBL 0021-9150 10.1016/j.atherosclerosis.2019.04.223 31051415 PMC6536321

[15] Lee M. W. et al., “Comprehensive intravascular imaging of atherosclerotic plaque *in vivo* using optical coherence tomography and fluorescence lifetime imaging,” Sci. Rep. 8(1), 14561 (2018). SRCEC3 2045-2322 10.1038/s41598-018-32951-9 30267024 PMC6162321

[16] Sherlock B. E. et al., “Simultaneous, label-free, multispectral fluorescence lifetime imaging and optical coherence tomography using a double-clad fiber,” Opt. Lett. 42(19), 3753–3756 (2017). OPLEDP 0146-9592 10.1364/OL.42.003753 28957119 PMC8951707

[17] Hsu P. S. et al., “Investigation of optical fibers for high-repetition-rate, ultraviolet planar laser-induced fluorescence of OH,” Appl. Opt. 52(13), 3108–3115 (2013). APOPAI 0003-6935 10.1364/AO.52.003108 23669781

[r18] Nuritdinov I. Masharipov K. Y. Doniev M., “Formation of radiation-induced defects in silica glasses at high irradiation temperatures,” Glass Phys. Chem. 29(1), 11–15 (2003). GPHCEE 1087-6596 10.1023/A:1022349524240

[19] Hillrichs G. et al., “Performance of low mode and single mode optical fibers for high peak power 355 nm laser radiation,” Proc. SPIE 7894, 78940Z (2011). PSISDG 0277-786X 10.1117/12.877836

[20] Frazão O. et al., “Stimulated Raman scattering and its applications in optical communications and optical sensors,” Open Opt. J. 3(1), 1–11 (2009). 10.2174/1874328500903010001

[21] Shrestha S. et al., “High-speed multispectral fluorescence lifetime imaging implementation for in vivo applications,” Opt. Lett. 35(15), 2558–2560 (2010). OPLEDP 0146-9592 10.1364/OL.35.002558 20680057 PMC4795163

[22] Serafino M. J. Applegate B. E. Jo J. A., “Direct frequency domain fluorescence lifetime imaging using field programmable gate arrays for real time processing,” Rev. Sci. Instrum. 91(3), 033708 (2020). RSINAK 0034-6748 10.1063/1.5127297 32260007 PMC7269681

[23] Jung W. et al., “Numerical analysis of gradient index lens-based optical coherence tomography imaging probes,” J. Biomed. Opt. 15(6), 066027 (2010). JBOPFO 1083-3668 10.1117/1.3523374 21198201 PMC3032234

[24] Duan C. et al., “Probe alignment and design issues of microelectromechanical system based optical coherence tomography endoscopic imaging,” Appl. Opt. 52(26), 6589–6598 (2013). APOPAI 0003-6935 10.1364/AO.52.006589 24085137

[25] Lee A. M. et al., “Fiber-optic polarization diversity detection for rotary probe optical coherence tomography,” Opt. Lett. 39(12), 3638–3641 (2014). OPLEDP 0146-9592 10.1364/OL.39.003638 24978556

[26] Kim W. et al., “Lensless, ultra-wideband fiber optic rotary joint for biomedical applications,” Opt. Lett. 41(9), 1973–1976 (2016). OPLEDP 0146-9592 10.1364/OL.41.001973 27128052 PMC6731063

[27] Brimacombe R. K. Taylor R. S. Leopold K. E., “Dependence of the nonlinear transmission properties of fused silica fibers on excimer laser wavelength,” J. Appl. Phys. 66(9), 4035–4040 (1989). JAPIAU 0021-8979 10.1063/1.343987 20531904

[28] Heimann J. et al., “Spectral UV losses in 355 nm pulsed laser delivery system at low temperatures,” J. Non-Cryst. Solids 376, 43–49 (2013). JNCSBJ 0022-3093 10.1016/j.jnoncrysol.2013.04.042

[29] Sherlock B. E. et al., “Multiscale, multispectral fluorescence lifetime imaging using a double-clad fiber,” Opt. Lett. 44(9), 2302–2305 (2019). OPLEDP 0146-9592 10.1364/OL.44.002302 31042209 PMC7539568

[30] Zuker M. et al., “Delta function convolution method (DFCM) for fluorescence decay experiments,” Rev. Sci. Instrum. 56(1), 14–22 (1985). RSINAK 0034-6748 10.1063/1.1138457

[31] Vishwasrao H. D. et al., “Conformational dependence of intracellular NADH on metabolic state revealed by associated fluorescence anisotropy,” J. Biol. Chem. 280(26), 25119–25126 (2005). JBCHA3 0021-9258 10.1074/jbc.M502475200 15863500

[32] Fang Q. et al., “Time-domain laser-induced fluorescence spectroscopy apparatus for clinical diagnostics,” Rev. Sci. Instrum. 75(1), 151 (2004). RSINAK 0034-6748 10.1063/1.1634354 PMC8920500 35291695

